# Integrating Chlorophyll a Fluorescence and Enzymatic Profiling to Reveal the Wheat Responses to Nano-ZnO Stress

**DOI:** 10.3390/plants12223808

**Published:** 2023-11-09

**Authors:** Shengdong Li, Yujia Liu, Zongshuai Wang, Tianhao Liu, Xiangnan Li, Peng Zhang

**Affiliations:** 1 Crop Research Institute, Shandong Academy of Agricultural Sciences, Jinan 250100, China; lsd01@163.com (S.L.); wzshuai0109@163.com (Z.W.); 2 Key Laboratory of Black Soil Conservation and Utilization, Northeast Institute of Geography and Agroecology, Chinese Academy of Sciences, Changchun 130102, China; liuyujia@iga.ac.cn (Y.L.); lixiangnan@iga.ac.cn (X.L.); 3 Engineering Laboratory for Ecoagriculture in Water Source of Liaoheyuan, Chinese Academy of Sciences, Liaoyuan 136200, China; liutianhao@iga.ac.cn

**Keywords:** carbon assimilation, nano-ZnO, net photosynthetic rate, total soluble sugar, wheat

## Abstract

It has been shown that increased concentrations of zinc oxide nanoparticles (nano-ZnO) in the soil are harmful to plant growth. However, the sensitivity of different wheat cultivars to nano-ZnO stress is still unclear. To detect the physiological response process of wheat varieties with different tolerance to nano-ZnO stress, four wheat cultivars (viz., cv. *TS1*, *ZM18*, *JM22*, and *LM6*) with different responses to nano-ZnO stress were selected, depending on previous nano-ZnO stress trials with 120 wheat cultivars in China. The results found that nano-ZnO exposure reduced chlorophyll concentrations and photosynthetic electron transport efficiency, along with the depressed carbohydrate metabolism enzyme activities, and limited plant growth. Meanwhile, the genotypic variation in photosynthetic carbon assimilation under nano-ZnO stress was found in wheat plants. Wheat cv. *JM22* and *LM6* possessed relatively lower Zn concentrations and higher leaf nitrogen per area, less reductions in their net photosynthetic rate, a maximum quantum yield of the PS II (F_v_/F_m_), electron transport flux per cross-section (ETo/CSm), trapped energy flux per cross-section (TRo/CSm), and total soluble sugar and sucrose concentrations under nano-ZnO stress, showing a better tolerance to nano-ZnO stress than wheat cv. *TS1* and *ZM18*. In addition, the chlorophyll a fluorescence parameters F_v_/F_m_, ETo/CSm, and TRo/CSm could be used to rapidly screen wheat varieties resistant to nano-ZnO stress. The results here provide a new approach for solving the issues of crop yield decline in regions polluted by heavy metal nanoparticles and promoting the sustainable utilization of farmland with heavy metal pollution.

## 1. Introduction

Nanoparticles (NPs) are used in a diverse range of industrial productions and daily life (e.g., cosmetic, medicine, and agriculture), and the range of applications is becoming increasingly widespread [1]. Thus, the release of NPs, which mostly contain heavy metals, into the environment is unavoidable, raising worldwide environmental issues [2]. The heavy metal NPs with surface structural and small-dimension effects cannot only attach themselves to most surfaces but also react with bio-molecules and are even penetrated into the interior of cells [3,4]. Previous studies have demonstrated that, when excessive NPs enter the plants, they can lead to the over-production of reactive oxygen species (ROS), depressed protein activity, and lipid peroxidation, hence affecting plant health [5,6]. In plant cells, heavy metal ions (NPs) can bind to the cysteine residue thiol group at the enzyme activity center, thus affecting enzyme activity [7]. NPs can also trigger severe oxidative bursts (overproduction of ROS), which can cause oxidative damage to proteins in chloroplasts, thereby decreasing the efficiency of photosynthetic carbon assimilation [8]. Additionally, NPs can depress the activities of enzymes (e.g., phosphoglucoisomerase (PGI), sucrose synthase (Susy), and aldolase (Ald)) in the tricarboxylic acid cycle and glycolysis process, thereby hindering the mitochondrial cell energy supply system [5].

A previous study documented that soil may be the main accumulation site for NPs; thus, most of crop will be exposed to NPs [9]. NPs can enter into plants via their roots, be transported to various organs, and affect photosynthesis and other metabolic processes [10]. Zinc oxide nanoparticles (nano-ZnO), one of most common heavy metal NPs, are widely applied to medicines, pigments, and batteries [11]. The unique nanostructures and nanoscale characteristics of nano-ZnO have attracted intense interest from the public and scientists over recent years and shown positive effects in agricultural production, such as promoting seed germination, plant development, alleviating abiotic stress, and improving plant resistance [12,13,14]. For example, the presence of nano-ZnO could improve antioxidant systems, speed up proline accumulation, and increase photosynthetic efficiency in tomato plants, indicating a positive effect of nano-ZnO on plant growth [15]. However, the negative impact of nano-ZnO cannot be ignored; past research has indicated that nano-ZnO has a dose effect relation to some extent [16]. A high dose of nano-ZnO can inhibit plant germination, chlorophyll biosynthesis, biomass accumulation, and generate ROS, endangering plant health [17]. A study found that the leaf gas exchange rates of Arabidopsis were notably decreased by more than 50% under nano-ZnO stress (300 mg L^−1^), which was related to the depression of the photosystem structure and chlorophyll synthesis genes expression [18]. Similarly, nano-ZnO stress (2000 mg L^−1^) could disrupt the adenosine triphosphate (ATP) synthesis and chloroplast-associated proteins, thereby reducing the chlorophyll content and interfering in photosynthetic carbon assimilation enzyme activities expression [19]. The effect of nano-ZnO is not only concentration-dependent but also varies among different cultivars of the same crop [20]. A previous study investigated the expression of six stress-related genes in three barley varieties (viz., *ZJU3*, *Golden Promise*, and *L23*) under nano-ZnO stress (300 mg L^−1^) and found that only one gene was upregulated; the other five were all downregulated [21]. Moreover, nano-ZnO treatment (150 mg kg^−1^) significantly reduced the chlorophyll b content of a soybean cultivar (cv, *Huachun 2*) but had no significant effect on the other soybean cultivar (cv, *Huachun 6*) [22].

The worldwide release of nano-ZnO has exceeded one million tons, and heavy metal NPs are bound to enter the atmosphere and water and eventually enrich in the soil [23]. The current research mainly focuses on the growth-promoting effects or improved resistance to abiotic stresses of nano-ZnO on wheat plants [24,25]. However, the negative impacts of nano-ZnO on wheat cannot be underestimated. In the present study, the physiological responses and performance of photosynthetic carbon assimilation of four wheat cultivars with different tolerances to nano-ZnO stress were investigated. The hypotheses were as follows: (i) The tolerance to nano-ZnO stress varies among different wheat cultivars; (ii) The response of the photosynthetic carbon assimilation process of wheat plants to nano-ZnO stress is related to the tolerance of nano-ZnO stress.

## 2. Results

### 2.1. Net Rate of Photosynthesis and SPAD Value

**Figure 1:**
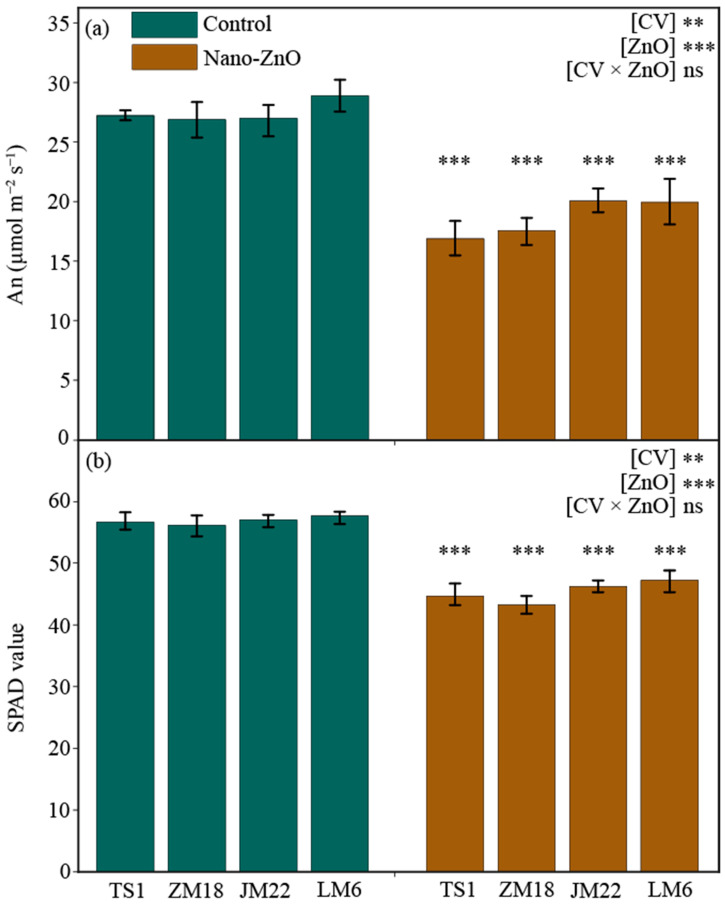
Net photosynthetic rate (An, (**a**)) and relative chlorophyll content (SPAD, (**b**)) in different wheat cultivars as affected by zinc oxide nanoparticles (nano-ZnO). Vertical bars indicate mean ± SE (*n* = 3). Non-nano-ZnO stress, Control; nano-ZnO stress, Nano-ZnO; wheat cultivars, *TS1*, *ZM18*, *JM22*, and *LM6*; **, *p* < 0.01; and ***, *p* < 0.001; ns, no significant difference.

After 55 days of treatment, both the net rate of photosynthesis (An) and relative chlorophyll content (SPAD) in leaves were significantly lower under nano-ZnO treatment than under the control, regardless of wheat cultivars (Figure 1). For each wheat cultivar, An and SPAD were decreased by more than 25% and 18%, respectively. The An of TSI (by 38.0%) and SPAD of *ZM18* (by 22.9%) showed the steepest decline, respectively (Table 1).

### 2.2. Chlorophyll Fluorescence Characteristics

**Figure 2:**
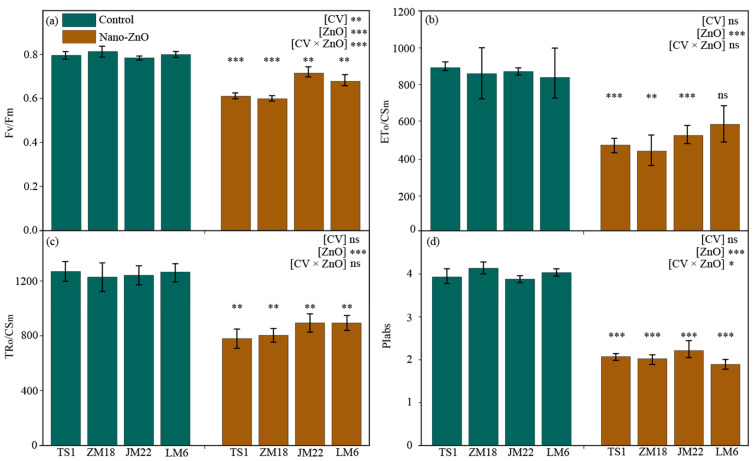
Changes in the maximum quantum yield of the PS II (Fv/Fm, (**a**)), electron transport flux per cross-section (ETo/CSm, (**b**)), trapped energy flux per cross-section (TRo/CSm, (**c**)), and performance index on absorption basis (PIabs, (**d**)) in different wheat cultivars as affected by zinc oxide nanoparticles (nano-ZnO). Vertical bars indicate mean ± SE (*n* = 3). Non-nano-ZnO stress, Control; nano-ZnO stress, Nano-ZnO; wheat cultivars, *TS1*, *ZM18, JM22*, and *LM6*; *, *p* < 0.05; **, *p* < 0.01; ***, and *p* < 0.001; ns, no significant difference.

Regardless the wheat cultivars, the maximum quantum yield of the PS II (F_v_/F_m_), electron transport flux per cross-section (ETo/CSm), trapped energy flux per cross-section (TRo/CSm), and performance index on absorption basis (PIabs) in wheat plants grown under nano-ZnO treatment were significantly lower than that under the control, except for ETo/CSm in *LM6* (Figure 2). The largest reduction in F_v_/F_m_, ETo/CSm, and PIabs occurred in *ZM18*, reaching 26.4%, 49.4%, and 51.0%, while TRo/CSm appeared in *TS1* (by 38.7%) (Table 1). The output of two-way ANOVA indicated that the F_v_/F_m_ and PIabs were significantly affected by the interaction of CV × ZnO (Figure 2a,d).

### 2.3. Rubisco and ATPase Activities

**Figure 3:**
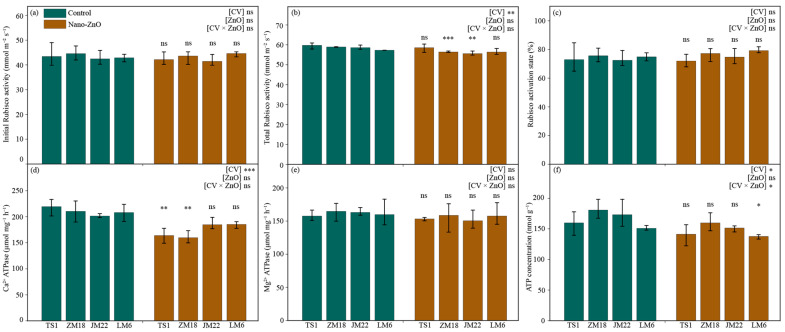
Activities of initial Rubisco (**a**) and total Rubisco (**b**), Rubisco activation (**c**), activities of Ca^2+^-ATPase (**d**) and Mg^2+^-ATPase (**e**), and ATP concentration (**f**) in different wheat cultivars as affected by zinc oxide nanoparticles (nano-ZnO). Vertical bars indicate mean ± SE (*n* = 3). Non-nano-ZnO stress, Control; nano-ZnO stress, Nano-ZnO; and wheat cultivars, *TS1*, *ZM18*, *JM22*, and *LM6*; *, *p* < 0.05; **, *p* < 0.01; and ***, *p* < 0.001; ns, no significant difference.

Compared with the control, the total Rubisco activities in *ZM18* and *JM22* were significantly decreased (by 4.2% and 5.2%, respectively) under the nano-ZnO treatment (Figure 3b; Table 1). Similar trends to total Rubisco activities were found in the Ca^2+^-ATPase activities in *TS1* and *ZM18*, as well as the ATP concentration of *LM6* (Figure 3d,f). Additionally, nano-ZnO stress depressed the initial Rubisco activities in *TS1*, *ZM18*, and *JM22* (by 2.9%, 2.2%, and 2.3%, respectively) and Mg^2+^-ATPase activities in each cultivar (by 2.6%, 3.8%, 7.8%, and 1.4%, respectively); it also increased the Rubisco activation in *ZM18*, *JM22*, and *LM6* (by 2.1%, 3.0%, and 5.8%, respectively), though the decrease or increase was not statistically significant (Figure 3a,c,e; Table 1). Additionally, the concentration of ATP was significantly affected by the interaction of CV × ZnO (Figure 3f).

### 2.4. Key Carbohydrate Metabolism Enzymes Activities

**Figure 4:**
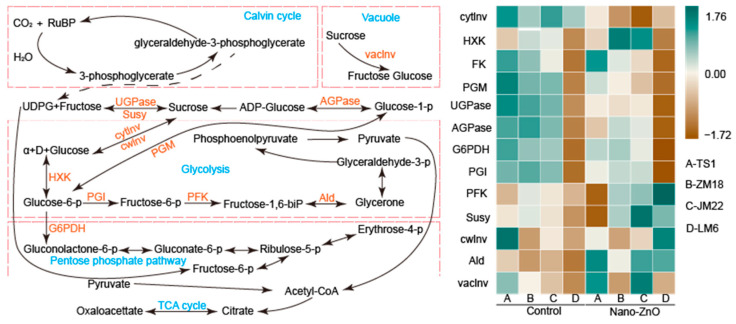
Heat map of key carbohydrate metabolism enzyme activities in different wheat cultivars as affected by zinc oxide nanoparticles (nano-ZnO). The difference in activity for a given enzyme among the different treatments was normalized and converted to a color scale. Vertical bars indicate mean ± SE (*n* = 3). Non-nano-ZnO stress, Control; nano-ZnO stress, Nano-ZnO; wheat cultivars, *TS1*, *ZM18*, *JM22*, and *LM6*; cytoplasmic invertase, cytInv; hexokinase, HXK; fructokinase, FK; phosphoglucomutase, PGM; UDP-glucose pyrophosphyorylase, UGPase; ADP-glucose pyrophosphorylase, AGPase; glucose-6-phosphate dehydrogenase, G6PDH; phosphoglucoisomerase, PGI; phosphofructokinase, PFK; sucrose synthase, Susy; cell wall invertase, cwInv; aldolase, Ald; and vacuolar invertase, vacInv.

The nano-ZnO treatment had different effects on the activities of key carbohydrate metabolism enzymes in these four wheat cultivars (Figure 4). For *TS1* plants, nano-ZnO treatment significantly limited the activities of UDP-glucose pyrophorylase (UGPase), phos-phoglucomutase (PGM), phosphoglucoisomerase (PGI), and ADP-glucose pyrophosphorylase (AGPase) as compared with the control. For *ZM18* plants, the activities of cytoplasmic invertase (cytInv), UGPase, PGI, and AGPase in plants grown under nano-ZnO treatment were lower than that under the control. For *JM22* plants, nano-ZnO treatment significantly decreased the activities of cytInv, UGPase, PGM, and AGPase but increased the activities of hexokinase (HXK) and aldolase (AId). The PGI, UGPase, and cytInv activities in *LM6* leaves were notably lower under nano-ZnO treatment than that under the control, while the trends of the activities of cell wall invertase (cwInv), phosphofructokinase (PFK), and Ald were exactly opposite.

### 2.5. Leaf N, Zinc, Sugars Concentrations, and Shoot and Root Dry Matters

**Figure 5:**
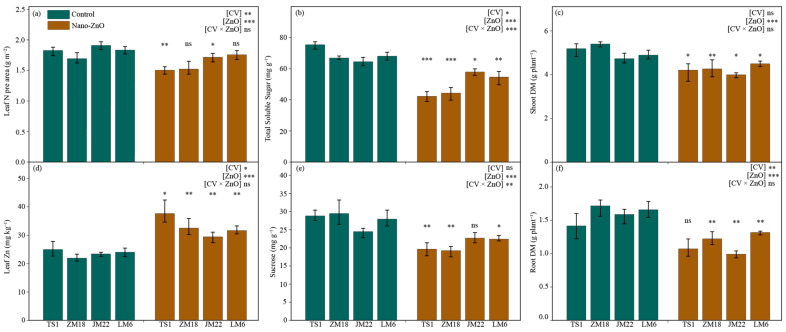
The concentrations of leaf nitrogen per area (**a**), leaf total soluble sugar (**b**), shoot dry matter (Shoot DM, (**c**)), leaf Zn (**d**), leaf sucrose (**e**), and root dry matter (Root DM, (**f**)) in different wheat cultivars as affected by zinc oxide nanoparticles (nano-ZnO). Vertical bars indicate mean ± SE (*n* = 3). Non-nano-ZnO stress, Control; nano-ZnO stress, Nano-ZnO; and wheat cultivars, *TS1*, *ZM18*, *JM22*, *LM6*; *, *p* < 0.05; **, *p* < 0.01; and ***, *p* < 0.001; ns, no significant difference.

Across all wheat cultivars, leaf nitrogen concentrations per area (leaf N) were depressed under nano-ZnO treatment as compared with the control, though statistical significance only showed in *TS1* (by 17.7%) and *JM22* (by 10.1%) (Figure 5a; Table 1). In contrast, the leaf zinc (Zn) concentration was higher under nano-ZnO treatment than that under the control in each wheat cultivar, and the increase in Zn concentration in *TS1* was the largest, reaching 50.5% (Figure 5d; Table 1).

The total soluble sugar (TSS) concentrations in leaves were significantly lower in all wheat cultivars under nano-ZnO treatment, compared with the control (Figure 5b). Similarly, the concentrations of sucrose in leaves were significantly decreased under ZnO treatment in *TS1*, *ZM18*, and *LM6* when compared to the control (Figure 5e). The maximum decrease in TSS and sucrose appeared in the *TS1* (by 43.9%) and *ZM18* (by 34.7%), respectively (Table 1). Additionally, the interactive effects of CV × ZnO were significant on the concentrations of TSS and sucrose (Figure 5b,e).

For all wheat cultivars, shoot dry matter was significantly depressed by nano-ZnO treatment, with a decrease of more than 15% compared to the control (except for *LM6*, which only decreased by 8.0%) (Figure 5c; Table 1). A similar trend appeared in the root dry matter in each wheat cultivar under Nano-ZnO treatment. Interestingly, the decline in all wheat cultivars exceeded 20%, though this was not significant in *TS1* (Figure 5f; Table 1).

## 3. Discussion

The toxic effects of Nano-ZnO on crop development and production are mostly due to disturbed photosynthesis, carbohydrate metabolism, and signal transduction [26,27]. A large body of studies reported that the reduction in chlorophyll contents and photosynthesis are the main symptoms of plant heavy metal (e.g., zinc) poisoning [4,21,28]. Consistent with this, the results here have shown that both An and SPAD values were notably depressed under Nano-ZnO stress (Figure 1), suggesting that the reduction in chlorophyll contents caused a decrease in photosynthesis and, hence, depressed plant performance. This was due to the fact that Nano-ZnO limited the expression of the photosystem structure and chlorophyll synthesis genes, resulting in the decrease in photosynthesis efficiency and the depression of chlorophyll biosynthesis [18]. The previous studies illuminated that the impact of NPs on photosynthesis was dose-dependent [17], and their effects varied in different plant species [29]. Here, the data of An showed a large genotypic variation, which was closely related to the tolerance to nano-ZnO stress in wheat. The reduction in SPAD caused by nano-ZnO stress did not differ obviously among these four wheat cultivars, suggesting that the damage of nano-ZnO stress to chlorophyll was similar in these cultivars.

It has been demonstrated that NPs in plant cells can interfere with the electron transport chain of chloroplasts, which may cause an increase in photoinhibition in plants [30]. The fast chlorophyll a fluorescence induction curve has been widely used to investigate the photosynthetic electron transport processes under various environmental changes [31,32,33]. A previous study showed a depressed electron transport efficiency and lower trapped energy flux in nano-ZnO-stressed wheat plants, in relation to non-stressed plants [8]. In the current study, nano-ZnO stress-induced changes in chlorophyll a fluorescence parameters were different among these wheat cultivars. Under nano-ZnO stress, except for ETo/CSm in *LM6*, the four chlorophyll a fluorescence parameters (viz., F_v_/F_m_, ETo/CSm, TRo/CSm, and PIabs) in all wheat cultivars were significantly decreased. Especially, the PIabs in *ZM18* was decreased by 51.0% in relation to the control (Figure 2), which was due to nano-ZnO damage to energy absorption that was more pronounced in *ZM18* than in other cultivars. Interestingly, the downward trend in F_v_/F_m_ in Nano-ZnO-susceptible cultivars (*TS1* and *ZM18*) was much higher than that in nano-ZnO-tolerant cultivars, indicating that F_v_/F_m_ was sensitive to the nano-ZnO stress and that chlorophyll a fluorescence can be used as a non-invasive tool to detect damage to plants under nano-ZnO stress. Further analysis on the derived parameters from the chlorophyll a fluorescence induction curve demonstrated that ETo/CSm and TRo/CSm showed higher reductions in two nano-ZnO-sensitive cultivars (*TS1* and *ZM18*) in relation to the nano-ZnO-tolerant cultivars (*JM22* and *LM6*) under nano-ZnO stress (Figure 2). Combined with An and SPAD values (Figure 1), this implies that nano-ZnO treatment (500 mg L^−1^) lead to a large number of zinc ions entering the central atoms of the chlorophyll, thereby forming Zn-chlorophyll, some of which were more stable than Mg-chlorophyll [34]. This phenomenon depressed the photosynthetic capture ability of Zn-chlorophyll, so as to trigger a collapse in photosynthesis [1].

Studies have reported that Rubisco initiates photosynthetic carbon assimilation through RuBP carboxylation [35]. The findings here show that Rubisco activation and initial total Rubisco activity and were not significantly changed by nano-ZnO stress (Figure 3a–c), which might be caused by the cultivar variations. In addition, the activities of Ca^2+^-ATPase and Mg^2+^-ATPase in chloroplasts play key roles in the conversion of light energy into stable chemical energy [36]. In this study, both Ca^2+^-ATPase and Mg^2+^-ATPase activities were inhibited via nano-ZnO treatment, though only the Ca^2+^-ATPase activities in *TS1* and *ZM18* were significantly decreased. It was suggested that the decreased Ca^2+^-ATPase and Mg^2+^-ATPase activities in wheat plants caused the inhibition of ATP synthesis, which might result in the depression of PS II (e.g., lowered F_v_/F_m_) and further promote the membrane lipid peroxidation of chloroplasts [37].

As the key regulator, the carbohydrate metabolism enzyme system is very sensitive to environmental fluctuations [38]. Here, the key carbohydrate metabolism enzyme activities were affected significantly by nano-ZnO treatment in leaves (Figure 4). Nano-ZnO exposure did not significantly affect sucrose synthase (Susy) and vacuolar invertase (vacInv) activities, which resulted in the decreased activities of UGPase and AGPase (only significant in *TS1* and *ZM18*), indicating that the synthesis processes of starch and sucrose were inhibited [39] (Figure 5b,e). In spite of the depressed activities of PGM (only significant in *TS1* and *JM22*) and PGI (not significant in *JM22*), the enhanced activities of fructokinase (FK) and PFK (only significantly in *JM22* and *LM6*) in leaves under nano-ZnO stress were similar to the previous study in barley; glycolysis and ATP synthesis are still inhibited due to the significant reduction in PGM and PGI [40]. Moreover, as a rate-limiting enzyme, the activity of glucose-6-phosphate dehydrogenase (G6PDH) was limited by nano-ZnO stress in each cultivar, suggesting that the pentose phosphate pathway in wheat plants was depressed by nano-ZnO stress.

The concentration of leaf N is often used as an indicator for the CO_2_ assimilatory capacity of crops, and carbon assimilation is dependent on leaf N [41]. For wheat plants, a common relationship has been found between the CO_2_ assimilation rate and leaf N for plants grown under varied conditions [42]. In the present study, nano-ZnO significantly reduced leaf N in four wheat cultivars by 4.2% to 17.7% and the highest and lowest reduction appeared in *TS1* and *LM6*, respectively (Figure 5a). This indicated that leaf N may be related to the tolerance of wheat plants to nano-ZnO stress. In addition, the SPAD value is usually used to monitor the leaf N status, depending on the relationship between SPAD readings and leaf N [43]. However, it should be noted that the trend of leaf N was inconsistent with that of SPAD among the four wheat cultivars under nano-ZnO stress. This suggested that the leaf N was better than SPAD for indicating the tolerance of wheat plants to nano-ZnO stress.

Compared with their respective control, the concentration of leaf Zn in the four cultivars increased by 25.8–50.5% under nano-ZnO treatment (Table 1). The two nano-ZnO-sensitive cultivars (*TS1* and *ZM18*) possessed a larger increase in leaf Zn concentration, while the nano-ZnO tolerant cultivars (*JM22* and *LM6*) showed relatively lower leaf Zn concentration (Figure 5d). It was indicated that the genotypic variation in uptake and accumulation of toxic Zn was related to the difference in nano-ZnO tolerance in wheat cultivars. The nano-ZnO can be easily absorbed through the lateral root junctions [44] and then transported and accumulated in specific subcellular locations such as cell vacuoles, nuclei, and plasmodesmata [45], thus altering plant physiological processes and plant growth [4,8,17]. The current results also confirmed this point, e.g., An, SPAD, and F_v_/F_m_ (Figure 1 and Figure 2).

Sugar not only acts as the prime carbon and energy sources for plant growth but also functions in stress resistance [46]. The sugar metabolism pathway is closely associated with the plant’s response to various environmental factors, such as cold, drought, and salt stress [47]. However, the effect of NPs on the sugar metabolism pathway in wheat has rarely been studied. Here, the concentrations of TSS and sucrose in leaves were significantly reduced by nano-ZnO stress in wheat plants (Figure 5b,e). Consistent with this, reduced TSS was observed in rice plants exposed to nano-TiO_2_ stress [48]. In the present study, the reductions in TSS and sucrose were larger in the nano-ZnO-susceptible cultivars than nano-ZnO-tolerant cultivars under nano-ZnO stress, suggesting that higher sugar levels may benefit tolerance to nano-ZnO stress and improve plant growth in wheat.

The negative effects of NPs on biomass accumulation have been reported in many crops, including soybean, wheat, and rice [1,8,49]. The shoot and root dry matters of all wheat cultivars were significantly decreased by nano-ZnO stress in this study (Figure 5). Combined with carbon assimilation and leaf Zn content in wheat plants, this indicates that the toxicity of nano-ZnO was most probably caused by the high levels of dissolved Zn [26]. Further study is needed to investigate whether the toxicity of nano-ZnO could be fully explained through the dissolution of Zn. Moreover, nano-ZnO-induced reductions in shoot and root dry matters were different among the four wheat cultivars. Compared with the control, the largest decrease caused by nano-ZnO in shoot dry matter was found in *ZM18*, while the largest decrease in root dry matter was in *JM22*. In addition, the nano-ZnO stress-induced reduction in root dry matter was higher than that in shoot DM for these four cultivars, indicating that the roots were more sensitive to nano-ZnO stress than shoots in wheat plants.

## 4. Materials and Methods

### 4.1. Experimental Materials and Design

Four wheat cultivars with different responses to nano-ZnO stress were selected, depending on previous nano-ZnO stress trials with 120 wheat cultivars. The wheat cv. *TS1* and *ZM18* are nano-ZnO-susceptible, and the wheat cv. *JM22* and *LM6* are nano-ZnO-tolerant. Eight seeds per wheat cultivar were sown in a plastic pot (8 L; height and diameter were 15 and 25 cm, respectively) and four strong sprouts of wheat were exposed to the treatment after the third leaf stage. Each pot was filled with 5 kg of clay soil moistened thoroughly with suspensions with 0 or 500 mg L^−1^ nano-ZnO (particle size < 50 nm; Dekedao nano Inc., Beijing, China). The soil contained 1.16 g kg^−1^ of total N and 33.1 and 129.6 mg kg^−1^ of P and K, respectively. Before planting, 1.2 g N, 0.75 g K, and 0.16 g of P were added to the soil in each pot. Each wheat seedling was developed in an artificial climate chamber at 26/16 °C (day/night). The photosynthetic active radiation (PAR) was set as >500 μmol m^−2^ s^−1^ over a 12 h photoperiod. At the 6-leaf stage, all measurements were applied to the plants from the control (viz., 0 mg L^−1^ nano-ZnO), while the nano-ZnO treatment (viz., 500 mg L^−1^ nano-ZnO) was applied using three replicates per treatment.

### 4.2. Gas Exchange and Chlorophyll a Fluorescence

After 55 days of nano-ZnO treatment (6-leaf stage), the last healthy and totally unfolded leaf was chosen to measure the An using a portable photosynthesis system (LI-6400, LI-Cor, Inc., Lincoln, NE, USA). The CO_2_ concentration and PAR in the leaf chamber were set to 400 μmol mol^−1^ and 1200 μmol m^−2^ s^−1^, respectively. Meanwhile, a chlorophyll meter (SAPD-502, Minolta, Osaka, Japan) was selected to measure the SPAD using the same leaf. After that, the leaf was used to test the fast chlorophyll a fluorescence induction curve via a plant efficiency analyzer (Pocket-PEA, Hansatech, Norfolk, UK). It should be noted that plants need to adapt to darkness for 30 min before measurement.

### 4.3. Rubisco and ATPase Activities

A total of 0.2 g fresh weight of leaf sample was ground into homogenate in the extraction buffer (40 mL), which comprised 1 mM EDTA, 10 mM β-mercaptoethanol, 10% polyvinylpyrrolidone (PVP), 1 mM MgCl_2_, and 50 mM Tris-HCl. Homogenate was centrifuged at 15,000× *g* for 15 min at 4 °C and the Rubisco activities were obtained using the supernatant. The initial and total Rubisco activities were measured as described by Wang et al. [49]. The activation of Rubisco was based on the ratio of initial Rubisco activity to total Rubisco activity for each sample. The ATPase activities (Ca^2+^-ATPase and Mg^2+^-ATPase) and ATP concentration were measured following the method of Zheng et al. [37].

### 4.4. Key Carbohydrate Metabolism Enzymes Activities

The 13 key carbohydrate metabolism enzymes activities in wheat leaves were applied according to the methods of Jammer et al. [50]. The hexokinase (HXK), aldolase (Ald), sucrose synthase (Susy), phosphoglucoisomerase (PGI), phosphofructokinase (PFK), phosphoglucomutase (PGM), pyrophosphorylase (AGPase), fructokinase (FK), glucose-6-phosphate dehydrogenase (G6PDH), and UDP-glucose pyrophorylase (UGPase) were determined in kinetic enzyme assays. The activities of cytoplasmic invertase (cytInv), vacuolar invertase (vacInv), and cell wall invertase (cwInv) were tested in the endpoint assays. The measurement was performed using an Epoch Take3 spectrophotometer (BioTek Instruments, Inc., Winosky, VT, USA) with a 96-well microtiter format.

### 4.5. Leaf Nitrogen per Area, Leaf Zinc, Sugars Concentrations, and Shoot and Root Dry Matters

The leaf N was determined using the micro Kjeldahl method, and the area of leaf sample was measured using a leaf area meter (LI-3100, Li-Cor Inc., Lincoln, NE, USA) to calculate the leaf N. The concentration of Zn in plant leaf samples was analyzed after high-pressure digestion with nitric acid (UltraClave III, MLS, Leutkirch, Germany) using inductively-coupled plasma optical emission spectrometry (ICP-OES 720, Varian, Palo Alto, CA, USA). The concentrations of total soluble sugar (TSS) and sucrose in leaf samples were measured using the anthrone reagents method. Shoot and root dry matters were obtained by drying the samples at 75 °C for 72 h.

### 4.6. Statistical Analysis

All the measured parameters had three replicates. A boxplot was used to test the homogeneity of variance of all data. Statistical significance between nano-ZnO and the cultivar was examined using two-way analysis of variance and covariance (ANOVA).

## 5. Conclusions

**Figure 6:**
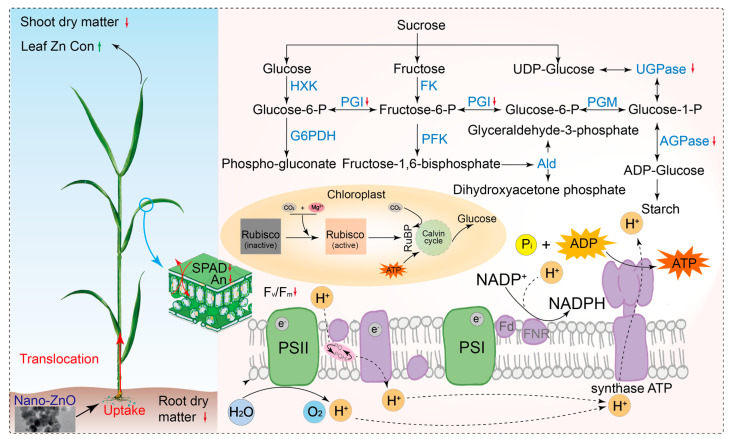
A comprehensive description of the response of wheat plants (e.g., *ZM18*) to nano-ZnO stress. The green up and red down arrows indicate positive and negative effects of nano-ZnO on the physiological processes of wheat plants. Hexokinase, HXK; phosphoglucoisomerase, PGI; glucose-6-phosphate dehydrogenase, G6PDH; fructokinasem, FK; phosphofructokinase, PFK; phosphoglucomutase, PGM; UDP-glucose, pyrophosphyorylase, UGPase; aldolase, Ald; ADP-glucose pyrophosphorylase, AGPase; net photosynthetic rate, An; stomatal conductance, g_s_; maximum quantum efficiency of photosystem II, F_v_/F_m_; photosystem I, PSI; photosystem II, PS II; ribulose-1,5-bisphosphate, RuBP; adenosine diphosphate, ADP; adenosine triphosphate, ATP; and nicotinamide adenine dinucleotide phosphate, NADPH.

Based on the pot experiment, nano-ZnO showed negative effects on photosynthetic carbon assimilation in wheat plants, as exemplified by the reduced chlorophyll concentration and photosynthetic electron transport efficiency. The different changes in the activities of carbohydrate metabolism enzymes implied that wheat plants control the response of glycolysis to nano-ZnO exposure, while the glycolysis regulation strategies varied across cultivars (Figure 6). Meanwhile, the changes in shoot and root dry matters showed that *LM6* might be one of the candidates for developing nano-ZnO tolerant wheat cultivars. In addition, the F_v_/F_m_, ETo/CSm, and TRo/CSm could be useful for rapidly screening wheat cultivars tolerant to nano-ZnO stress. The above results might be a great help to solve the problem of yield reduction in regions polluted by heavy metal NPs.

## Figures and Tables

**Table 1:** Effect of zinc oxide nanoparticles (nano-ZnO) on tested parameters in different wheat cultivars.

Cultivars	An	SPAD	F_v_/F_m_	ET_o_/CS_m_	TR_o_/CS_m_	PI_abs_	IRA	TRA	RA	Ca^2+^A	Mg^2+^A	ATPc	Leaf N	Leaf Zn	TSS	SR	Shoot DM	Root DM
*TS1*	−38.0%	−21.1%	−23.4%	−47.5%	−38.7%	−47.3%	−2.9%	−2.0%	−1.1%	−25.3%	−2.6%	−11.7%	−17.7%	+50.5%	−43.9%	−31.7%	−18.8%	−24.2%
*ZM18*	−34.7%	−22.9%	−26.4%	−49.4%	−34.6%	−51.0%	−2.2%	−4.2%	+2.1%	−24.1%	−3.8%	−11.5%	−10.0%	+48.5%	−33.7%	−34.7%	−21.0%	−28.7%
*JM22*	−25.7%	−18.8%	−8.8%	−40.1%	−28.1%	−43.0%	−2.3%	−5.2%	+3.0%	−8.3%	−7.8%	−12.5%	−10.1%	+25.8%	−10.2%	−7.5%	−15.6%	−37.4%
*LM6*	−31.0%	−18.1%	−15.3%	−30.6%	−29.4%	−53.2%	+4.1%	−1.6%	+5.8%	−11.0%	−1.4%	−8.7%	−4.2%	+31.8%	−19.8%	−19.7%	−8.0%	−20.6%

Note: Net photosynthetic rate, An; relative chlorophyll content, SPAD; maximal photochemical efficiency, F_v_/F_m_; electron transport flux per cross-section, ET_o_/CS_m_; trapped energy flux per cross-section, TR_o_/CS_m_; performance index on an absorption basis, PI_abs_; initial Rubisco activity, IRA; total Rubisco activity, TRA; Rubisco activation, RA; Ca^2+^-ATPase activity, Ca^2+^A; Mg^2+^-ATPase activity, Mg^2+^A; ATP concentration, ATPc; leaf N per area content, Leaf N; leaf zinc content, Leaf Zn; leaf total soluble sugar concentration, TSS; leaf sucrose concentration, SR; shoot dry matter, Shoot DM; and root dry matter, Root DM. “+” and “−“ indicate positive and negative effects of nano-ZnO, respectively, on the physiological parameters of wheat plants. (*n* = 3).

## Data Availability

Data are contained within the article.
